# Protective role of black garlic water extract in kidney injury induced by cisplatin in mice

**DOI:** 10.1186/s12906-025-05178-1

**Published:** 2025-12-15

**Authors:** Shin-Mei Lee, Yu-Ting Cheng, Ming-Chu Tsai, Liang-Ju Chen, Hung-Chuan Pan, Yu-Chin Lin, Ching-Wen Chang, Yi-Ching Tsai, Chia-Yun Tsai, Wen-Chin Lee, De-Wei Lai

**Affiliations:** 1 Department of Food Science, National Quemoy University, Kinmen, Taiwan; 2 Department of Natural Biotechnology, Nanhua University, Chiayi, Taiwan; 3 Department of Nursing, Da-Yeh University, Changhua, Taiwan; 4 Department of Post-Baccalaureate in Nursing, Da-Yeh University, Changhua, Taiwan; 5 Department of Health-Business Administration, Hungkuang University, Taichung, Taiwan; 6 Neurological Institute, Taichung Veterans General Hospital, Taichung, Taiwan; 7 Department of Medical Research, Taichung Veterans General Hospital, Taichung, Taiwan; 8 Ph.D. Program in Translational Medicine, Rong Hsing Research Center for Translational Medicine, National Chung Hsing University, Taichung, Taiwan; 9 Doctoral Program in Biotechnology Industrial Management and Innovation, National Chung Hsing University, Taichung, Taiwan; 10 Department of Medicinal Botanicals and Foods on Health Applications, College of Biotechnology and Bioresources, Da-Yeh University, Changhua, Taiwan; 11 Department of Cosmetic Applications and Management, MacKay Junior College of Medicine, Nursing, and Management, Taipei, Taiwan; 12 Precision gene and cell center, Chang Bing Show Chwan Memorial Hospital, Changhua, Taiwan; 13 Department of Internal Medicine, Division of Nephrology, Chang-Bing Show Chwan Memorial Hospital, Changhua, Taiwan; 14 Department of Post-Baccalaureate Medicine, College of Medicine, National Chung Hsing University, Taichung, Taiwan

**Keywords:** Acute kidney injury, Cisplatin, Black garlic, Antioxidant enzyme

## Abstract

**Background:**

Acute kidney injury (AKI) is a common and serious complication of cisplatin chemotherapy in cancer patients, with no effective treatment currently available. Oxidative stress and renal tubular damage are key contributors to its pathogenesis. This study aimed to investigate the antioxidant and renoprotective effects of black garlic water extract in a cisplatin-induced AKI mouse model.

**Methods:**

Mice were randomly divided into six groups: Control, Cisplatin only (20 mg/kg), black garlic extract pretreatment at 50 or 100 mg/kg followed by cisplatin (Cis + B50, Cis + B100), amifostine pretreatment (200 mg/kg) as positive control (Cis + A200), and black garlic extract only (100 mg/kg). Black garlic extract was characterized for its sulfur compound content and antioxidant potential.

**Results:**

Black garlic contained significantly higher levels of S-allyl-L-cysteine (191.2 ± 32.87 µg/g) than raw garlic (20.7 ± 0.8 µg/g) and effectively delayed low-density lipoprotein oxidation. Pretreatment with black garlic extract reduced cisplatin-induced weight loss, renal index elevation, and tubular damage. Antioxidant enzyme activities, including superoxide dismutase, catalase, glutathione peroxidase, and glutathione reductase, were significantly increased in the Cis + B100 and Cis + B50 groups.

**Conclusions:**

Black garlic extract confers protection against cisplatin-induced AKI by enhancing renal antioxidant defenses and mitigating oxidative stress-related damage. These findings support its potential as a complementary approach for preventing nephrotoxicity during cisplatin therapy.

**Supplementary Information:**

The online version contains supplementary material available at 10.1186/s12906-025-05178-1.

## Introduction

 Cisplatin (Cis) is widely used to treat various cancers; however, its nephrotoxicity significantly impacts the patient willingness to undergo chemotherapy [1, 2]. Two weeks of Cis administration significantly increased serum biochemical markers, including blood urea nitrogen (BUN) and creatinine, and decreased creatinine clearance, indicating renal dysfunction [3]. Therefore, mitigating the side effects without compromising the efficacy of chemotherapy is important to improve the patient adherence to treatment.

Cis, a first-line chemotherapeutic agent, induces acute kidney injury (AKI) asthe most severe adverse effect. This drug generates excessive reactive oxygen species (ROS) in renal tubular cells, leading to oxidative stress, which is a key contributor to nephrotoxicity [4]. Although mitochondria activate various antioxidant enzymes (e.g., catalase, superoxide dismutase [SOD], glutathione peroxidase [GPx], and glutathione reductase [GRd]) to neutralize ROS, prolonged exposure depletes these enzymes, reducing the intracellular glutathione levels [5, 6]. ROS accumulation ultimately causes mitochondrial dysfunction, cell death, and renal impairment [7]. Animal studies have demonstrated that endogenous antioxidant depletion plays a more critical role in oxidative damage than ROS accumulation, underscoring the physiological significance of antioxidants as oxidative injury biomarkers [8]. Notably, dietary antioxidants mitigate oxidative stress. For example, nutrient-rich fruits and vegetables contain vitamin C, lycopene, and lutein, which are associated with cardiovascular protection, neuroprotection (e.g., Alzheimer’s disease prevention), reduced oxidative damage in various diseases (e.g., cancer), and enhanced cellular repair mechanisms [9]. However, specific antioxidant effects of black garlic against Cis-induced oxidative stress remain unclear.

Black garlic is produced via raw garlic biotransformation, which increases the levels of bioactive compounds, including antioxidants, total polyphenols, water-soluble organosulfur compounds (e.g., S-allyl-L-cysteine[SAC]), and Maillard reaction products [10, 11]. S-allyl-L-cysteine (SAC), a highly bioavailable garlic-derived sulfur compound, is primarily distributed in the plasma, liver, and kidneys, and exerts potent antioxidant, anti-aging, immunomodulatory, cardioprotective, hepatoprotective, anti-allergic, and anticancer effects [12, 13]. Additionally, raw garlic ameliorates chemotherapy-induced side effects [14, 15]. However, its role as an antioxidant stabilizer in Cis therapy remains unclear. Amifostine has been approved in clinical practice for reducing cisplatin-induced nephrotoxicity in patients with advanced ovarian cancer, as well as for alleviating xerostomia caused by radiotherapy in head and neck cancer patients, which often limits treatment options. Its protective mechanism is generally attributed to scavenging free radicals and preserving cell membranes [16]. In this study, amifostine was used as a positive control to evaluate the renoprotective effects of black garlic extract in the animal model.

Preclinical studies have provided direct in vitro and in vivo evidence that garlic-derived preparations and specific garlic organosulfur compounds potentially protect renal tissue from oxidative injury and can attenuate Cis-induced nephrotoxicity. In rodent models, administration of aged garlic extract (AGE), which is enriched in water-soluble organosulfur compounds such as SAC, significantly reduced Cis-induced increases in serum creatinine and BUN, improved histopathological renal changes, and restored antioxidant enzyme activities (including SOD and catalase) compared with Cis alone [17]. Black garlic (produced by prolonged aging of raw garlic) contains higher concentrations of SAC and other Maillard-reaction products with demonstrated antioxidant and anti-inflammatory activity compared with raw garlic, and recent nutritional/toxicology studies report that black garlic extracts can protect hepatic and renal cells from diverse toxic insults in animal models [18]. These observations provide a biologically plausible rationale for testing black garlic extract specifically in Cis-induced AKI models: its enriched SAC content and other antioxidant constituents are expected to restore depleted endogenous antioxidants and reduce oxidative damage in renal tubular cells, thereby attenuating Cis-driven renal dysfunction.

Cis-induced AKI is caused by drug accumulation in renal tubules, leading to DNA damage, oxidative stress, and apoptosis, irreversibly progressing to the chronic kidney disease. In this study, we examined the effects of the black garlic extract on renal tissues and antioxidant enzyme activities in mice to clarify its nephroprotective effects. By identifying the protective agents against Cis-induced renal injury.

## Materials and methods

### Reagents

Cisplatin was purchased from Sigma (St. Louis, MO, USA). Glutathione peroxidase (GPx), glutathione reductase (GRd), and the GSH/GSSG assay kit were obtained from Cayman Chemicals (Ann Arbor, MI, USA). The SOD assay kit was obtained from Faith Technology (Taichung, Taiwan). Ethylenediaminetetraacetic acid (Na2-EDTA) and magnesium chloride (MgCl2⋅6H2O) were purchased from Showa (Tokyo, Japan).

### Garlic sample Preparation

Fresh garlic bulbs (Allium sativum L.) were purchased from Yunlin County, Taiwan. This variety is cultivated in Kinmen, where garlic is typically planted around October and harvested in April, followed by natural sun-drying for approximately one month. The sun-dried raw garlic bulbs were directly used for this study. Black garlic was processed from the same variety by a certified local manufacturer (EVERSTRONG BIOTECH. CO., LTD.). Bulbs with a diameter of 6–8 cm were selected, and after removing the black garlic peels, the black garlic samples were subjected to extraction. For extract preparation, raw or black garlic were homogenized with distilled water (25 ± 2℃) (100 rpm; 16 h) at a ratio of 1:3 (w/v). The homogenate was passed through an 80-mesh (Whatman No.1 filter paper) sieve to obtain the filtered solution, which was subsequently freeze-dried for 3 days to produce a dry raw and black garlic extract powder. Preparation of black garlic extract powder flow chart as showed in the Supplementary Fig. 1.

### Quantitative analysis of S-Allyl-L-Cysteine (SAC) and Diallyl disulfide (DADS)

SAC and diallyl disulfide, the sulfur-containing compounds in garlic, were analyzed as described by Arnault et al. [19]. A Thermo RP-C18 column (4.6 mm i.d., 150 mm, 5 μm) was used. The mobile phase started with 100% 10 mM heptanesulfonic acid (pH 2.0, adjusted with orthophosphoric acid) in water for 4 min, followed by a linear gradient to ACN/10 mM heptanesulfonic acid (7:3, v/v) over 5 min, and then to 100% ACN over 25 min. The flow rate was 1 mL/min, and detection was at 208 nm.

### Copper ion-induced low-density lipoprotein (LDL) oxidation lag time

LDL (770200-1, Kalen Biomedical) was diluted in 5.0 mM phosphate-buffered saline (PBS; pH 7.0) to a concentration of 0.15 mg/mL. Following the method described by Esterbauer et al. (1992) [20], 125–135 µL of PBS and 5–20 µL of test samples were added to 96-well quartz microplates (HELLMA 730.009-Q), followed by 100 µL LDL and 10 µL of 125 µM CuSO₄, reaching 250 µL total volume. Absorbance at 232 nm was measured every 10 min for 12 h using an ELISA reader (µQuant, Bio-Tek). Lag time was determined by identifying the intersection between the baseline and the tangent of the propagation phase on the oxidation curve.

### Experimental animals

48 Male BALB/c mice, six-week-old were obtained from the National Laboratory Animal Center (Taipei, Taiwan) and maintained at 21 ± 2 °C temperature under 65 ± 5%humidity, with an automatic timer controlling the light/dark cycle (12 h each; light period from 7:00 AM to 7:00 PM; dark period from 7:00 PM to 7:00 AM). The mice were weighed and randomly assigned to cages after statistical analyses. The mice were provided ad libitum access to water and diet (LabDiet 5001 chow, Young Li). Randomization and allocation: Animals were randomly assigned to experimental groups by an investigator not otherwise involved in animal handling or outcome assessment. Randomization lists were generated using a computer random number generator (simple randomization with equal allocation/or block randomization with block size = 4), and animals were allocated sequentially according to the randomized list. Cage locations and order of interventions were balanced across groups to minimize environmental confounders. Blinding: Personnel who performed the interventions were not involved in data collection and analysis. All samples (blood, serum, tissue) and histological slides were labeled with coded identifiers such that investigators performing biochemical assays, histological scoring, and image analysis were blinded to group allocation. The code was broken only after the data were finalized.

### Anesthesia and euthanasia

All animal experiments were conducted in accordance with the institutional guidelines for the care and use of laboratory animals and were approved by the Institutional Animal Care and Use Committee (IACUC) of Chang Bing Show Chwan Memorial Hospital, under protocol number #113,008. Prior to sacrifice, mice were anesthetized with inhalation overdose of isoflurane. Adequate anesthesia was confirmed by the absence of pedal reflex. Subsequently, euthanasia was performed by carbon dioxide inhalation, followed by cervical dislocation to ensure death before tissue collection.

### Establishment of acute kidney injury model

Next, an AKI mouse model was established as described by Mitazaki et al. [21], with slight modifications. Six-week-old male BALB/c mice were acclimatized for one week and intraperitoneally injected with Cis (20 mg/kg body weight) to induce renal injury, a dosage widely applied in rodent models of cisplatin-induced nephrotoxicity [22].

### Evaluation of the protective effects of black Garlic extract on acute kidney injury

After one week of acclimatization, seven-week-old male BALB/c mice were divided into six groups: Control, 20 mg/kg Cis (Cis), 50 mg/kg black garlic extract pretreatment + Cis (Cis + B50), 100 mg/kg black garlic extract pretreatment + Cis (Cis + B100), 200 mg/kg amifostine pretreatment + Cis (Cis + A200; positive control), and black garlic extract alone (100 mg/kg) groups. The amifostine dose (200 mg/kg) was selected based on previous studies demonstrating its protective role against cisplatin-induced renal injury in mice [23]. An AKI mouse model was established as described by Mitazaki et al., with some modifications. Then, the AKI model mice were intraperitoneally injected with Cis (20 mg/kg body weight) at 72 h to induce renal injury. Black garlic extract-treated mice were orally administered black garlic extract for eight consecutive days. On day 6, black garlic extract was administered 30 min before the intraperitoneal injection of Cis (20 mg/kg body weight) to induce injury. On day 9, the mice were sacrificed and body weight, kidney weight, and kidney index (kidney weight/body weight × 100) were measured.

### Histological staining of kidney tissue

After euthanization using carbon dioxide, mouse kidney tissues were collected, fixed with10% formalin, embedded in paraffin, sectioned, and stained with hematoxylin and eosin (ab245880, Abcam, UK). Tissue damage was assessed based on previously reported classification criteria [24]. The tubular dilatation and necrosis scores in medulla and cortex were estimated at 400× magnification using 10 randomly selected fields.

### Serum expression of BUN and creatinine

Serum was cryopreserved immediately after isolation. BUN (E-BC-K183-M; Elabscience, USA) and creatinine (ab65340; Abcam, UK) levels were measured using ELISA kits, and absorbance at 450 nm was recorded with a plate reader. Concentrations were calculated based on standard curves.

### GPx activity assay

GPx activity was assessed using a commercial kit (E-BC-K096-M; Elabscience, USA). After adding tissue homogenate and H₂O₂, absorbance at 340 nm was recorded for 5 min, and activity was calculated using E₃₄₀ = 6220 M⁻¹cm⁻¹.

### GRd activity assay

GRd activity was measured using an ELISA kit (ECGR-100; BioAssay Systems, USA). MgCl₂, GSSG, NADPH, and phosphate buffer (pH 7.0) were mixed with homogenates per kit instructions. The decrease in NADPH at 340 nm was recorded over 5 min.

### SOD activity assay

SOD activity was determined using a kit (706002; Cayman Chemicals). Superoxide was generated via a xanthine/xanthine oxidase system, and the rate of cytochrome c-[Fe(III)] reduction to cytochrome c-[Fe(II)] was used to calculate activity, expressed as U/mg protein.

### Measurement of GSH and GSSG antioxidant enzyme activities

For GSH, 200 µL of tissue homogenate was mixed with trichloroacetic acid, incubated on ice for 5 min, and centrifuged. The supernatant was reacted with tris/EDTA buffer and DTNB, and absorbance at 412 nm was measured (E₄₁₂ = 13600 M⁻¹cm⁻¹).

For GSSG, 250 µL homogenate was treated with 0.04 M N-ethylmaleimide and NaOH, then reacted with o-phthaldehyde and NaOH. Fluorescence was measured at Ex = 420 nm and Em = 350 nm.

### Catalase activity assay

Tissues (20 mg) were homogenized in cold PBS using a Dounce homogenizer at 4 °C. Catalase activity was determined using a kit (E-BC-K031-M; Elabscience, USA) with absorbance read at 450 nm and quantified from standard curves.

### Statistical analysis

Data are represented as the mean ± standard error of the mean. Statistical analyses were conducted as previously described [25, 26]. Data are presented as mean ± SD (or median and interquartile range for non-normal data). Normality was assessed by Shapiro–Wilk test. For comparisons among ≥ 3 groups, one-way ANOVA followed by Tukey’s post hoc test was applied when data satisfied the normality assumption, whereas the Kruskal–Wallis test followed by Dunn’s test was used for non-normal data. For two-group comparisons, either a two-tailed Student’s t-test (two-tailed) was used as appropriate. Repeated-measures data were analyzed by two-way repeated measures ANOVA with correction for sphericity. Categorical variables were compared using Fisher’s exact test or χ² test where suitable. Statistical analyses were performed using GraphPad Prism (version 6.0). A p-value *< 0.05* was considered statistically significant. Details of statistical tests and sample sizes are indicated in the figure legends or Methods.

## Results

### Effects of black garlic on the body weight and kidney index of mice with Cis-induced nephrotoxicity

**Fig. 1:**
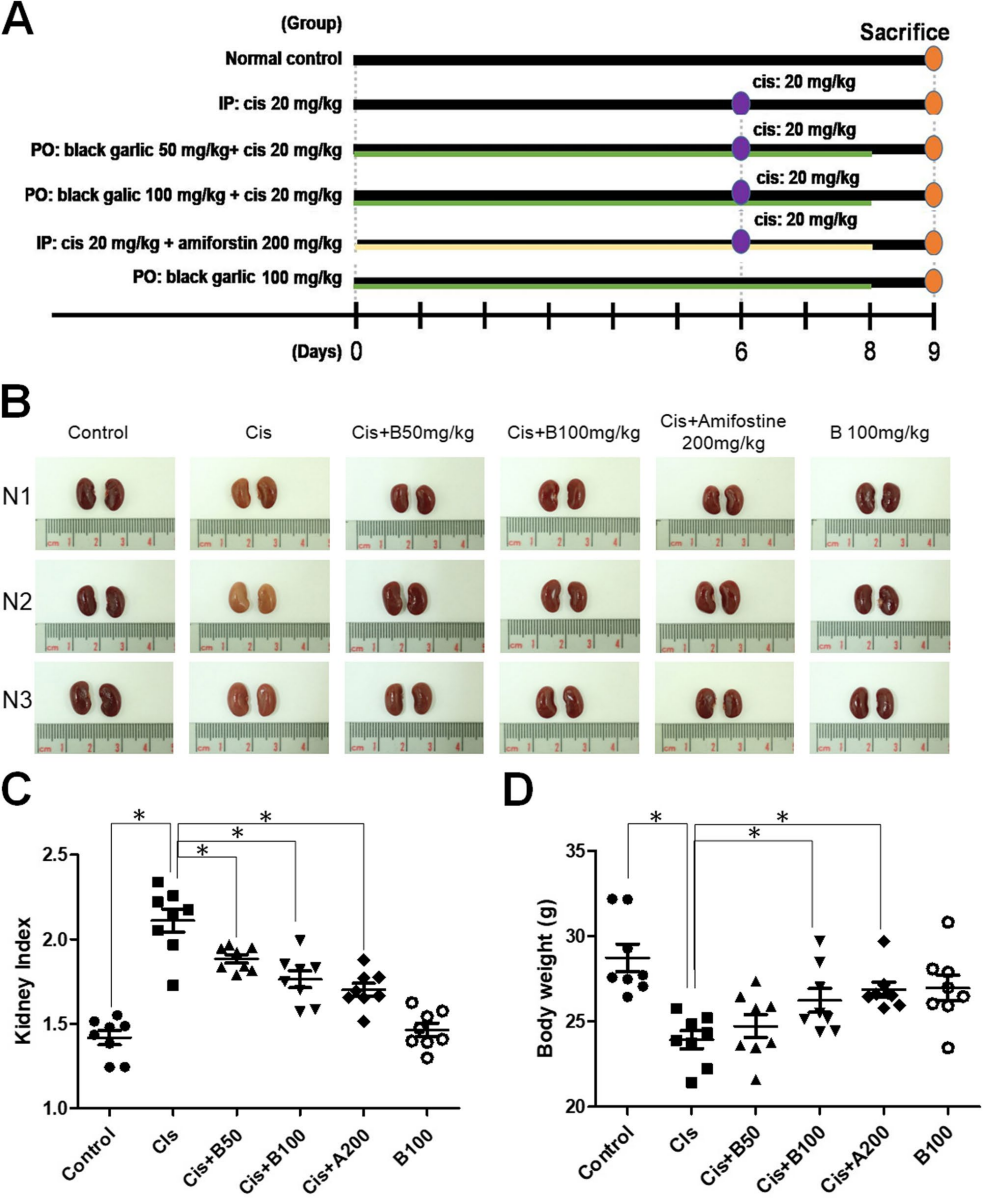
Experimental design and evaluation of kidney injury in alltreatment groups. **A** Schematic representation of the experimental design. Male BALB/c mice were divided into six groups: Normal control, cisplatin (Cis; 20 mg/kg), Cis with 200 mg/kg amifostine (Cis + A200), Cis with 50 mg/kg black garlic extract (Cis + B50), Cis with100 mg/kg black garlic extract (Cis + B100), and 100 mg/kg black garlic extract alone (B100). All treatments were administered via intraperitoneal (IP) injections or oral gavage (PO). After Cis was administered on day 6 for treat, and all mice were sacrificed on day 9. **B** Representative kidney morphology images of three individual mice (N1, N2, and N3) per group.**C** Kidney index (kidney weight/body weight × 100) indifferent groups. **D** Body weight changes in different groups. Data are represented as the mean ± SEM (*n* ≥ 6 per group).Statistical significance was indicated as *p < 0.05

To evaluate the protective effects of black garlic extract against Cis-induced nephrotoxicity, we examined the changes in kidney index and body weight in all treatment groups (Fig. 1). Garlic extract powder was prepared by homogenizing raw and black garlic with distilled water, filtering, and freeze-drying the solution, as detailed in Supplementary Fig. 1. Moreover, black garlic, which is rich in S-allyl-L-cysteine 191.2 ± 32.87 µg/g compared to 20.7 ± 0.8 µg/g in raw garlic (Table 1), and exhibits a prolonged LDL-C oxidation lag time (Table 2), further confirms that the black garlic produced by the preparation system possesses potent antioxidant properties. Macroscopically, kidneys of the cisplatin-treated group were yellow and slightly swollen, where as those of the black garlic-treated group showed recovery and resembled those of the control group (Fig. 1B). Cis significantly increased the kidney index compared to that in the control group, causing renal swelling and damage. However, co-treatment with Cis + B50 or Cis + B100 and Cis + A200 significantly alleviated these effects. Notably, black garlic extract alone (B100) did not alter the kidney index compared to that in the control group (Fig. 1C). Body weight was markedly reduced in the Cis group; however, co-administration of black garlic extract and amifostine significantly reduced weight loss (Fig. 1D), exerting protective effects against renal injury. These results highlight the renoprotective effects of black garlic against Cis-induced AKI.

**Table 1:** Extraction rates and functional compositions of Raw and black Garlic water extracts

	Raw garlic	Black garlic
Water extraction rate (%)	19.8%	57.5%
Sulfur compounds (µg/g extract)		
S -allyl-L-cysteine (SAC)	20.7 ± 0.8	191.2 ± 32.9*
Diallyl disulfide (DADS)	16.2 ± 0.5	11.4 ± 2.4

**Table 2:** Effects of Raw and black Garlic water extracts on copper ion-induced low-density lipoprotein (LDL) oxidation lag time

Garlic Sample	LDL-C lag time (min)
Control group (PBS)	58.0
Raw garlic	82.0
Black Garlic	396.0

### Cis-induced histopathological changes in kidneys and black Garlic protective effects

**Fig. 2:**
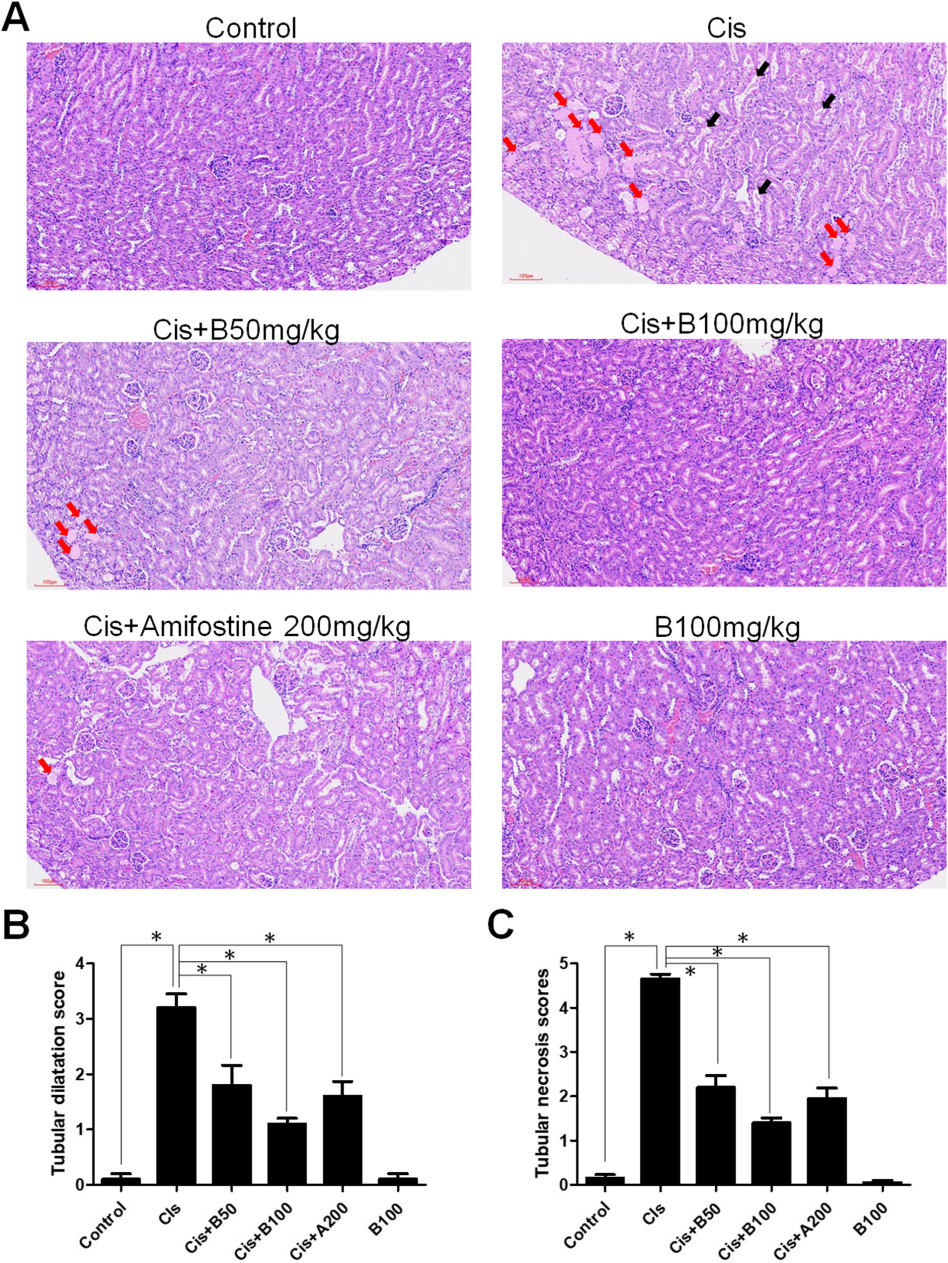
Histological analysis of kidney injury in all treatment groups. **A** Representative hematoxylin and eosin (H&E)-stained kidney sections of each experimental group. Control group exhibited normal renal morphology, whereas Cis group exhibited significant tubular damage, including tubular dilatation (red arrows) and tubular necrosis (black arrows). However, treatment with 50 mg/kg (Cis + B50) or 100 mg/kg (Cis + B100) black garlic extract and amifostine (Cis + A200) alleviated the kidney injury. Notably, B100 group showed normal renal architecture. Scale bar, 100 μm. **B** Quantification of tubular dilatation scores in different groups. **C** Quantification of tubular necrosis scores in different groups. Data are represented as the mean ± SEM (*n* ≥ 6 per group). Statistical significance was indicated as *p < 0.05. Cis: Cis-treated group; Cis + B50: Cis-treated + 50 mg/kg black garlic-treated group; Cis + B100: Cis-treated + 100 mg/kg black garlic-treated group; Cis + A200: Cis-treated + 200 mg/kg amifostine-treated group; B100 alone: 100 mg/kg black garlic-treated group without Cis treatment)

Histological analysis via hematoxylin and eosin staining was performed to further assess the kidney damage and black garlic protective effects (Fig. 2A). Control group exhibited normal renal architecture, whereas Cis group exhibited extensive tubular damage characterized by tubular dilation (black arrows), epithelial cell detachment, and necrosis (red arrows). However, these pathological changes were significantly ameliorated in the Cis + B50, Cis + B100, and Cis + A200 groups, with reduced tubular injury and preserved renal architecture. Notably, B100 group showed no apparent renal damage. Quantitative analysis revealed that tubular dilation and necrosis were significantly elevated in the Cis group but markedly reduced in the black garlic- and amifostine-treated groups (Fig. 2B and C). These results suggest that black garlic protects kidneys by alleviating the Cis-induced renal tubular damage.

### Black garlic-induced renal function marker improvement

**Fig. 3:**
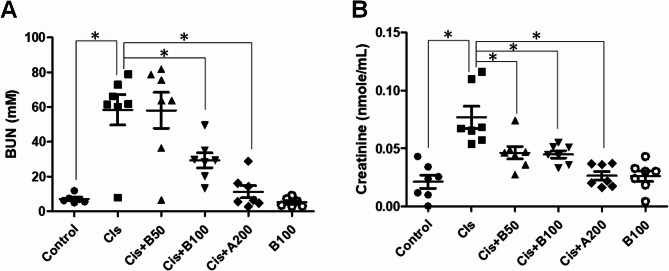
Effects of different treatments on the renal function markers in mice with Cis-induced nephrotoxicity. **A** Blood urea nitrogen (BUN) levels in different experimental groups. **B** Serum creatinine levels in different experimental groups. Data are represented as the mean ± SEM (*n* ≥ 6 per group). Statistical significance was indicated as *p < 0.05. Cis: Cis-treated group; Cis + B50: Cis-treated + 50 mg/kg black garlic-treated group; Cis + B100: Cis-treated + 100 mg/kg black garlic-treated group; Cis + A200: Cis-treated + 200 mg/kg amifostine-treated group; B100 alone: 100 mg/kg black garlic-treated group without Cis treatment)

To examine the nephroprotective effects of black garlic, we measured the serum levels of the key renal function indicators, BUN and creatinine (Fig. 3A and B). Cis significantly increased the BUN and creatinine levels compared to those in the control group, causing severe renal dysfunction. However, co-treatment with black garlic (Cis + B50 or Cis + B100) and amifostine (Cis + A200) significantly reduced these marker levels, thereby improving the renal function. B100 group maintained normal BUN and creatinine levels, further confirming the safety of the black garlic extract. These findings suggest that black garlic effectively mitigates Cis-induced renal toxicity and preserves the kidney function.

### Black Garlic alleviatesoxidative stress and restores the antioxidant enzyme activities in mice with Cis-induced nephrotoxicity

**Fig. 4:**
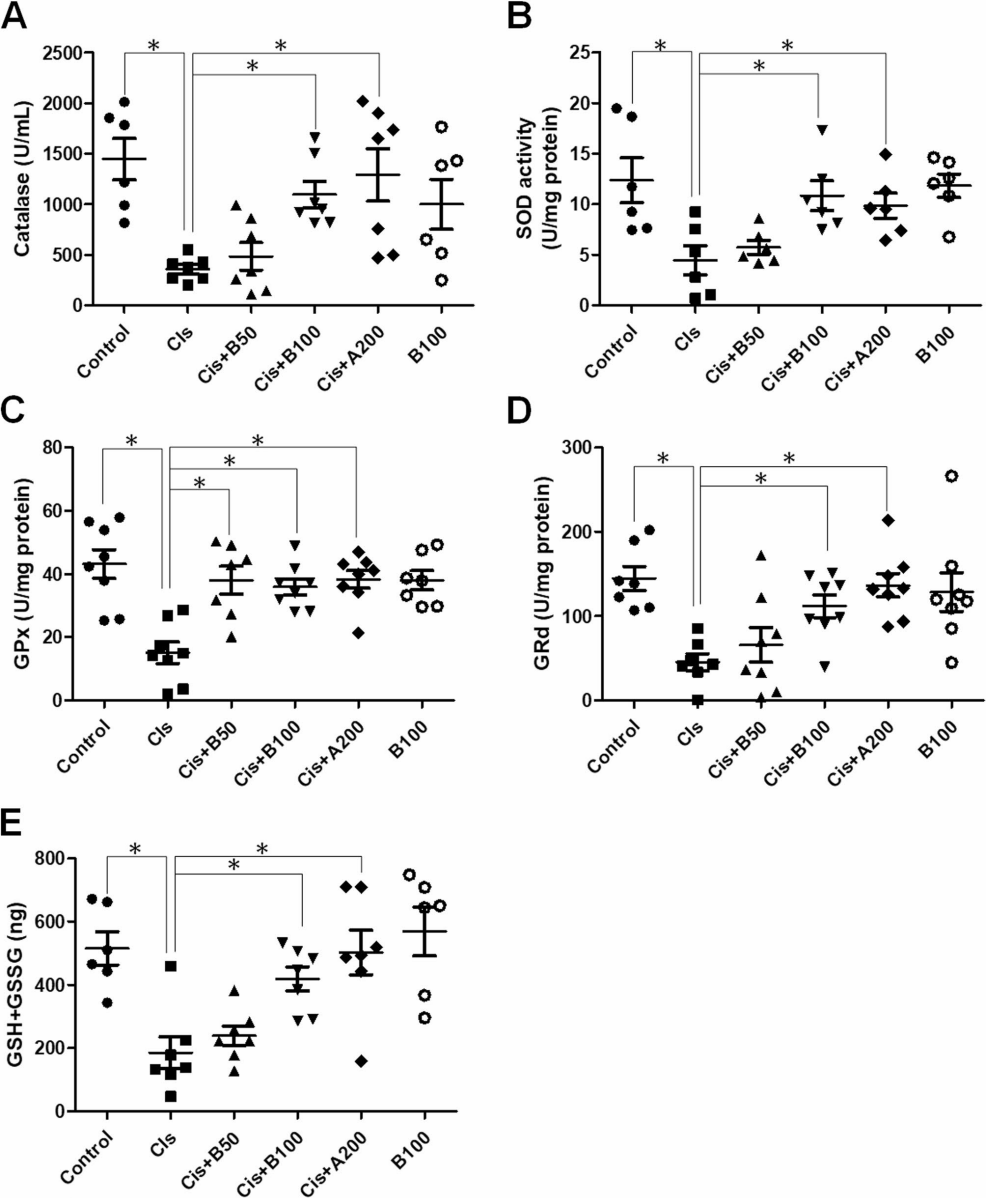
Effects of different treatments on the antioxidant enzyme activities and glutathione levels in all experimental groups. **A** Catalase activity (U/mL). **B** Superoxide dismutase (SOD) activity (U/mg protein). **C** Glutathione peroxidase (GPx) activity (U/mg protein). **D** Glutathione reductase (GRd) activity (U/mg protein). **E** Total glutathione (GSH + GSSG) level (ng). Data are represented as the mean ± SEM (*n* ≥ 6 per group). *p < 0.05, determined via analysis of variance (ANOVA), followed by post-hoc analysis. Cis: Cis-treated group; Cis + B50: Cis-treated + 50 mg/kg black garlic-treated group; Cis + B100: Cis-treated + 100 mg/kg black garlic-treated group; Cis + A200: Cis-treated + 200 mg/kg amifostine-treated group; B100 alone: 100 mg/kg black garlic-treated group without Cis treatment)

To evaluate the effects of black garlic on oxidative stress and antioxidant defense mechanisms in mice with Cis-induced nephrotoxicity, we measured the activities of key antioxidant enzymes, including catalase (Fig. 4A), SOD (Fig. 4B), GPx (Fig. 4C), and GRd (Fig. 4D), and total glutathione (GSH + GSSG) levels(Fig. 4E). Cis significantly reduced the catalase, SOD, GPx, and GRd activities (*p* < 0.05) and total glutathione levels (*p* < 0.05) compared to those in the control group. However, black garlic mitigated these effects in a dose-dependent manner. Specifically, B50 and B100 groups showed significant restoration of antioxidant enzyme activities and total glutathione levels compared to the Cis group (*p* < 0.05). Therefore, black garlic enhances the antioxidant defense responses and alleviates the Cis-induced oxidative damage in renal tissues.

### Black Garlic pre-treatment protects against cisplatin-induced nephrotoxicity

**Fig. 5:**
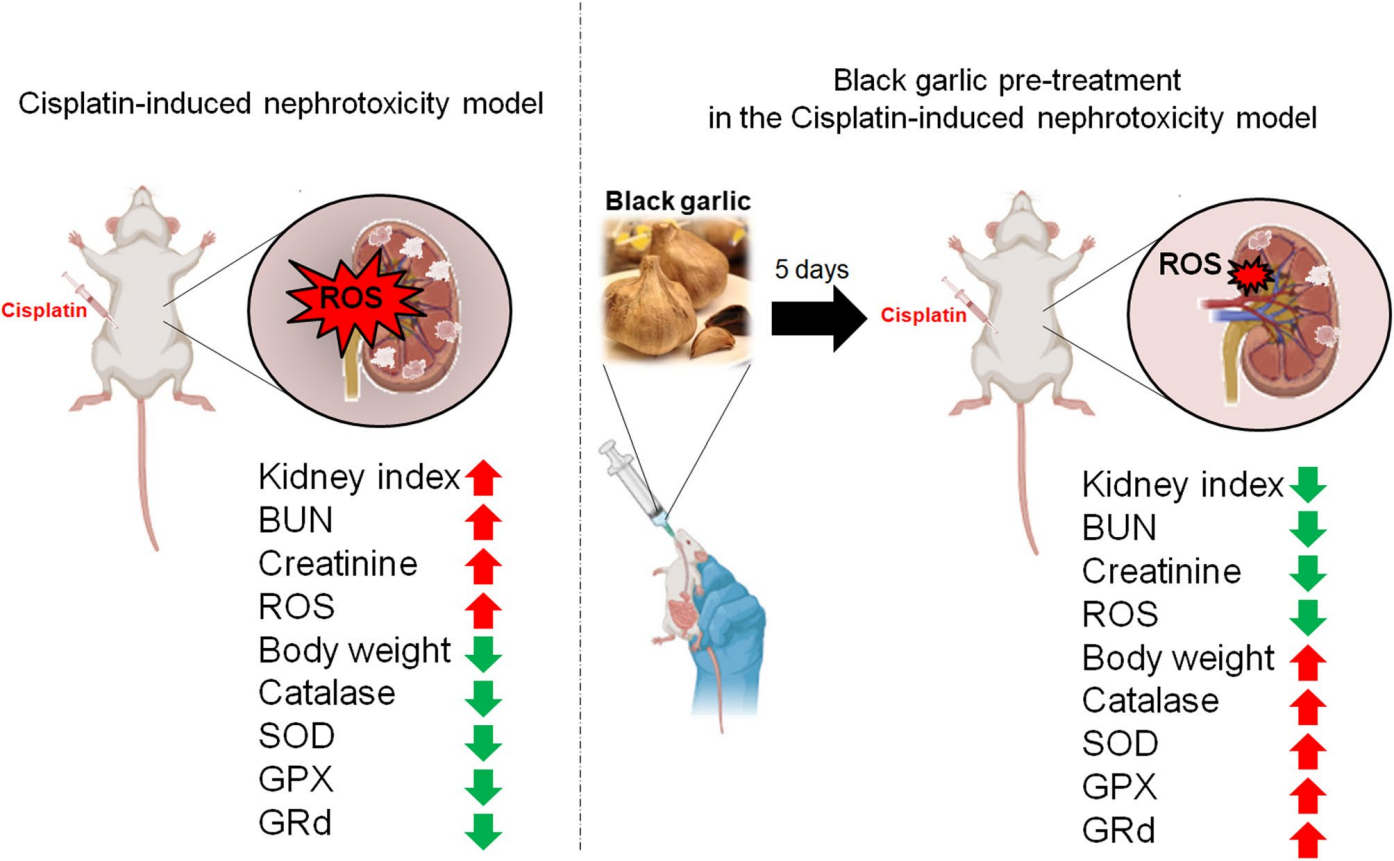
Protective effects of black garlic pretreatment against Cis-induced nephrotoxicity. Left panel illustrates the Cis-induced nephrotoxicity in mice, leading to increased ROS production in the kidneys and elevated kidney index and BUN and creatinine levels. Additionally, Cis decreased the body weight and antioxidant enzyme activities, including catalase, SOD, GPx, and GRd activities. Right panel shows the effects of black garlic pretreatment for five days prior to Cis administration; black garlic pretreatment alleviated nephrotoxicity by reducing the ROS levels, kidney index, and BUN and creatinine levels and enhancing the body weight and antioxidant enzyme activities. Upward and downward arrows indicate the increase and decrease in the corresponding parameters, respectively. The working model was created using BioRender (https://BioRender.com)

To further confirm the protective effects of black garlic, we examined its effect on Cis-induced nephrotoxicity. Cis significantly increased the kidney index and BUN, creatinine, and ROS levels but decreased the body weight and antioxidant enzyme activities. However, black garlic pretreatment for five days prior to Cis administration and subsequent co-treatment for three days significantly alleviated these nephrotoxic effects. Specifically, black garlic reduced the kidney index and BUN, creatinine, and ROS levels and restored the body weight and catalase, SOD, GPx, and GRd activities (Fig. 5). Therefore, black garlic preconditioning plays crucial roles in preserving the renal function and reducing the Cis-induced nephrotoxicity-associated oxidative damage.

## Discussion

Cis is a widely used chemotherapeutic agent; however, its dose-limiting nephrotoxicity remains a major clinical challenge, with no effective protection strategy currently available. Despite extensive research, pharmacological interventions to mitigate Cis-induced renal damage have shown limited success [27], necessitating the identification of alternative protective agents. This study highlighted the potential of black garlic to alleviate Cis-induced nephrotoxicity, suggesting it as a promising natural antioxidant against chemotherapy-associated renal complications.

Cis-induced nephrotoxicity is marked by oxidative stress, inflammation, and apoptosis, leading to tubular injury, elevated kidney index, and impaired renal function [28, 29]. Consistent with prior reports, Cis increased BUN, creatinine, and ROS levels, and induced tubular dilation, epithelial detachment, and necrosis, highlighting the role of oxidative stress in Cis-induced kidney injury [4]. In this study, black garlic supplementation significantly ameliorated these effects by enhancing antioxidant defenses and reducing oxidative damage. Bioactive compounds in black garlic, such as SAC and polyphenols, possess strong free radical-scavenging abilities [11, 30], and may activate key signaling pathways like the Nrf2 pathway to regulate antioxidant enzyme expression [31]. Additionally, black garlic reduced pro-inflammatory cytokines, including TNF-α and IL-6, thereby alleviating inflammation-induced renal damage [32].

Black garlic’s antioxidant effects have been shown in various disease models, underscoring its role in mitigating oxidative stress-related injury [33, 34]. It enhances antioxidant activity and decreases lipid peroxidation in conditions such as diabetic nephropathy and ischemia–reperfusion injury [35]. Previous studies have shown that aged garlic extract attenuates cisplatin-induced nephrotoxicity by enhancing antioxidant defenses and reducing lipid peroxidation [36]. Similarly, other natural antioxidants, such as curcumin, resveratrol, and quercetin, have demonstrated renoprotective effects through mechanisms involving Nrf2 activation, ROS suppression, and mitochondrial protection [37]. Our findings are consistent with these reports, as black garlic extract restored antioxidant enzyme activity and reduced oxidative stress in cisplatin-treated mice. Notably, unlike aged garlic, black garlic contains higher levels of sulfur-containing compounds, including S-allyl-L-cysteine and diallyl disulfide, which may provide additional benefits by supporting glutathione synthesis and stabilizing mitochondrial function. These unique phytochemical properties suggest that black garlic extract could offer advantages over other natural antioxidants, thereby underscoring the novelty of the present study. In addition, amifostine, a clinically approved cytoprotective agent, served as the positive control in this study. The amifostine-treated group demonstrated significant protection against cisplatin-induced renal injury, consistent with previous reports attributing its effects to free radical scavenging and stabilization of cellular membranes [38]. Including amifostine as a comparator allowed us to benchmark the efficacy of black garlic extract against an established nephroprotective agent, thereby strengthening the interpretation of our findings. Overall, these findings emphasize the potential of natural antioxidants as adjuvants to mitigate chemotherapy-induced toxicity without compromising efficacy.

Key antioxidant enzymes—SOD, GPx, GRd, and catalase—are vital for defending against chemotherapy-induced oxidative stress [39]. SOD converts superoxide radicals into hydrogen peroxide, which is further detoxified by catalase and GPx, thus reducing oxidative load in renal tissues. Cis treatment significantly suppressed these enzymes, aggravating oxidative injury [40]. In contrast, black garlic supplementation reversed these reductions, suggesting Nrf2-mediated activation of antioxidant defenses. Moreover, black garlic modulates mitochondrial function and apoptosis by adjusting the Bcl-2/Bax ratio and inhibiting caspase activation [41]. These mechanisms together support its nephroprotective potential. A potential limitation of our study is that the nephroprotective effect of black garlic extract is largely attributed to its antioxidant properties. Since the anticancer efficacy of cisplatin partly relies on ROS generation, it remains possible that concomitant antioxidant activity might attenuate its antitumor action. However, previous studies have reported inconsistent findings regarding the interaction between antioxidants and cisplatin, and certain constituents of black garlic, such as S-allyl-L-cysteine, have also been suggested to exert direct antitumor effects [42, 43]. Further investigations using tumor-bearing models are warranted to clarify whether black garlic extract compromises, preserves, or even enhances cisplatin’s anticancer efficacy. In addition, this study has several other limitations. First, only a single animal model was employed, and the sample size was relatively small, which may restrict the generalizability of the findings. Second, although our data suggest that the protective effect is associated with enhanced antioxidant defenses, we did not directly investigate molecular mechanisms such as the Nrf2 signaling pathway. Third, no data were collected on long-term outcomes or potential interactions with cisplatin’s anticancer efficacy in vivo. Addressing these limitations in future studies will provide a more comprehensive understanding of the therapeutic potential of black garlic.

From a translational perspective, the current study is limited to preclinical findings, and several issues remain to be addressed before clinical application. The optimal dosage and treatment regimen of black garlic extract for humans are not yet established, and potential drug–drug interactions with cisplatin should be carefully evaluated, given that both efficacy and toxicity may be influenced. Furthermore, future studies incorporating patient-derived samples or models will be critical to validate our observations and to better bridge the gap between experimental data and clinical practice. In conclusion, this study demonstrates that black garlic effectively protects against Cis-induced nephrotoxicity by enhancing antioxidant responses, reducing oxidative and histological damage, and preserving renal function. Pretreatment with black garlic provided superior protection, highlighting its potential as a prophylactic strategy. These findings position black garlic as a promising natural antioxidant for managing chemotherapy-related renal complications.

## Supplementary Information

Supplementary Material 1. Supplementary Figure 1. Preparation of black garlic extract powder. Black garlic cloves were homogenized with distilled water at a ratio of 1:3 (w/v). The homogenate was passed through an 80-mesh sieve to obtain the filtered solution, which was subsequently freeze-dried for 3 days to produce a dry black garlic extract powder.

## Data Availability

All relevant study data are included in the article and available from the corresponding author on reasonable request.

## Abbreviations

AKI: Acute kidney injury
BUN: Blood urea nitrogen
ELISA: Enzyme-linked immunosorbent assay
GPx: Glutathione peroxidase
GRd: Glutathione reductase
GSH/GSSG: Reduced glutathione/oxidized glutathione
LDL: Low-density lipoprotein
Nrf2: Nuclear factor erythroid 2-related factor 2
PBS: Phosphate-buffered saline
ROS: Reactive oxygen species
SAC: S-allyl-L-cysteine
SOD: Superoxide dismutase
