# Supplementary material for: Protective role of black garlic water extract in kidney injury induced by cisplatin in mice
Source: BMC Complement Med Ther. 2025 Dec 15;25:440. doi: 10.1186/s12906-025-05178-1 (PMC12706886; doi:10.1186/s12906-025-05178-1)
Supplement: Supplementary file 1 — Supplementary Material 1. Supplementary Figure 1. Preparation of black garlic extract powder. Black garlic cloves were homogenized with distilled water at a ratio of 1:3 (w/v). The homogenate was passed through an 80-mesh sieve to obtain the filtered solution, which was subsequently freeze-dried for 3 days to produce a dry black garlic extract powder. [file 12906_2025_5178_MOESM1_ESM.docx]

***Supplementary Information***

**
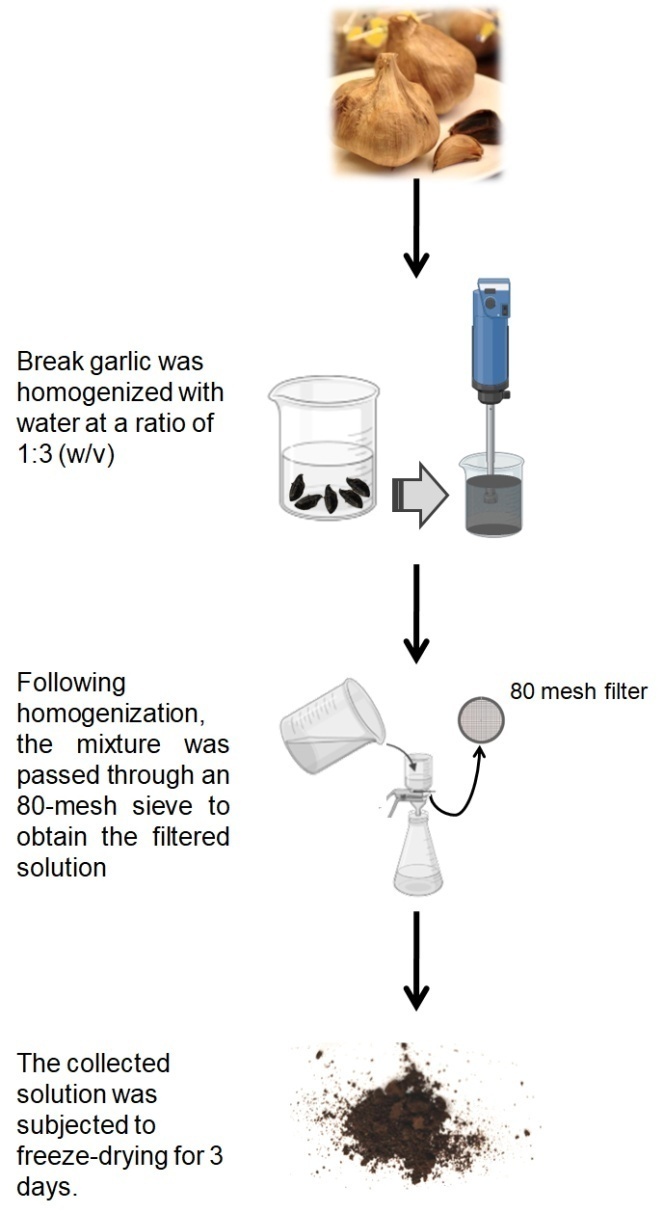
**

**Supplementary Figure 1. Preparation of black garlic extract powder.**

Black garlic cloves were homogenized with distilled water at a ratio of 1:3 (w/v). The homogenate was passed through an 80-mesh sieve to obtain the filtered solution, which was subsequently freeze-dried for 3 days to produce a dry black garlic extract powder.
